# Imagery-related eye movements in 3D space depend on individual differences in visual object imagery

**DOI:** 10.1038/s41598-022-18080-4

**Published:** 2022-08-19

**Authors:** Sandra Chiquet, Corinna S. Martarelli, Fred W. Mast

**Affiliations:** 1 Department of Psychology, University of Bern, 3012 Bern, Switzerland; 2 Faculty of Psychology, UniDistance Suisse, 3900 Brig, Switzerland

**Keywords:** Psychology, Human behaviour

## Abstract

During recall of visual information people tend to move their eyes even though there is nothing to see. Previous studies indicated that such eye movements are related to the spatial location of previously seen items on 2D screens, but they also showed that eye movement behavior varies significantly across individuals. The reason for these differences remains unclear. In the present study we used immersive virtual reality to investigate how individual tendencies to process and represent visual information contribute to eye fixation patterns in visual imagery of previously inspected objects in three-dimensional (3D) space. We show that participants also look back to relevant locations when they are free to move in 3D space. Furthermore, we found that looking back to relevant locations depends on individual differences in visual object imagery abilities. We suggest that object visualizers rely less on spatial information because they tend to process and represent the visual information in terms of color and shape rather than in terms of spatial layout. This finding indicates that eye movements during imagery are subject to individual strategies, and the immersive setting in 3D space made individual differences more likely to unfold.

## Introduction

When we recall detailed visual information from memory, we tend to move our eyes despite the fact that there is nothing to look at. Such eye movements have been shown to reinstate the spatial layout of previously inspected images^1–10^. For example, Spivey and Geng found that participants tend to re-fixate the spatial location of previously inspected stimuli, when they were questioned about visual details (i.e., color and orientation) in a subsequent recall task^11^ Experiment^2^. This effect, known as *looking at nothing*, is well established in healthy subjects and has been found when objects were encoded on 2D computer screens, even then when spatial memory was not probed in the recall task^8,12–16^. In a recent study we were able to demonstrate that participants looked back to relevant locations also in a seated immersive three-dimensional (3D) setting^15^.

It has been proposed that eye movements to absent objects are triggered by integrated memory representations^17,18^: During encoding, different features including spatial location are combined into a coherent representation. During recall, the spatial location is reactivated and triggers eye movements towards empty but relevant locations and thereby serves to facilitate memory retrieval of other object-related features (i.e., visual, linguistic, and conceptual). There is ample empirical support for this proposition, indeed, several studies suggest that spatial location is encoded and maintained automatically (e.g.,^19^) and some findings suggest that eye movements to absent objects plays a beneficial role for memory^8,12,13,20^, although some researchers reported inconclusive results^11,14,16^. Moreover, findings from studies on visual working memory support the idea that spatial location serves as mental index for object representations^21^ and that spatial location supports the integration of visual features (i.e., color and shape) into coherent memory representations^22–24^.

Interestingly, several studies showed remarkable differences in eye movements. For example, eye movement dispersion during imagery of simple visual patterns^10^ and of complex images^1,25^ have been found to vary between individuals and some people tend to spatially constrain their fixations around the location of initial gaze position during memory retrieval of previously seen objects^15,26^. Moreover, Kinjo et al. found comparable memory performance between participants who moved their eyes and those who remained with their eyes on the center of the screen, thus suggesting an individual trade-off between the benefits and costs of using eye movements during visual imagery and visual memory^26^. On the one hand, eye movements may aid memory retrieval as they serve to reinstate the spatial index associated with previously inspected visual information^4,8,12^. On the other hand, eye movements can induce processing costs; they are time-consuming^27^, require the activation of motor programs and can lead to interference with concurrent perceptual information from the external world^28^. This may be especially the case when the relative location of a target object is embedded in a 3D spatial layout when compared to a target presented on a blank screen in front of the participants (see^15^). By examining eye movements in an immersive 3D setting we can investigate in a more natural environment how individuals differentially engage the oculomotor system and spatial information in visual imagery of previously seen objects.

Recent research on visual scene imagery suggests that vividness of an internal representation is related to the degree of overlap between the neural activation involved during perceptual encoding and during imagery^3,29^. Furthermore, the reinstatement of neural activation has been shown to correlate with fixation reinstatement^3^. However, Gurtner et al. did not find a link between individual differences in creating vivid images (as measured with the Vividness of Visual Imagery Questionnaire, VVIQ^30^) and spatial correspondence of eye movements during imagery and perception of complex pictures (i.e., faces, landscapes, and art pictures)^2^. It is noteworthy that the VVIQ focuses on the ability to imagine visual scenes in terms of clarity, richness and resemblance to perceptual experience without considering how individuals process and represent visual information. Interestingly, scores in the VVIQ have been shown to correlate with self-reported measures of object-imagery ability, however not with self-reported measures of spatial imagery ability^31,32^.

Indeed, previous research has provided support for the distinction between object imagery versus spatial imagery on the level of individual differences (e.g.,^33^). For example, Blajenkova et al. developed a questionnaire (Object Spatial Imagery Questionnaire, OSIQ) to assess individual differences in imagery abilities^31^. The questionnaire identified two types of individuals in terms of their ability to process and represent visual information: Individuals who score high in the self-reported object imagery scale, called *object visualizers.* They process and represent information predominantly in terms of visual properties, such as color, shape, and texture. By contrast, individuals who score high in the self-reported spatial imagery scale, called *spatial visualizers,* process and represent objects and scenes predominantly in terms of their locations, motion, and spatial relations^34^. Scores in the self-reported object imagery scale of the OSIQ correlated with measures on object imagery (i.e., the ability to generate pictorial objects^34^) whereas scores on the spatial imagery scale have been shown to correlate with the ability to create, maintain, and inspect spatial representations^35^. Furthermore, Johansson et al. found that higher scores on the spatial imagery scale were negatively correlated with the overall dispersion of eye gaze patterns during visualization of complex pictures^25^. It remains unclear, however, how individual differences in imagery abilities are related to the involvement of spatial location in visual imagery of previously inspected objects. Eye movement reinstatement during imagery of single objects may relate to different processes than eye movements during imagery of complex scenes with interdependent features within a spatial layout. Within the scope of an integrated memory model^17,18^ spatial visualizers could rely predominantly on the object’s location to achieve vivid and detailed images whereas object visualizers process internal representations more holistically (see^34^) and may construct vivid and colorful images without looking at spatial locations of previously inspected stimuli.

**Figure 1:**
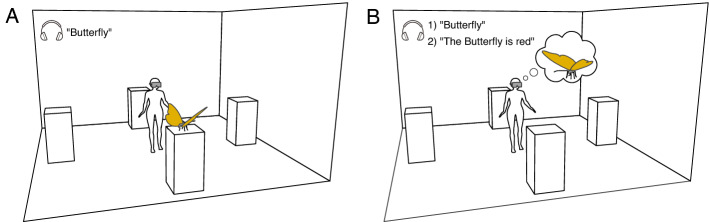
Schematic illustration of the virtual environment (**A**) during encoding and (**B**) during recall. During encoding, each trial started with the title followed by the appearance of the object either in front, to the right, to the left or behind the participants. During recall, participants were cued by the title (1) to visualize the objects they had encoded before (image generation), and they evaluated a statement (true/false) (2) about visual details of the object (image inspection).

In this study, we used immersive virtual reality (IVR) to investigate whether eye fixation patterns during visual imagery of previously seen virtual objects in 3D space depend on how individuals process and represent visual information. During perceptual encoding, participants were shown 28 virtual objects in sequence, each with a unique title. The objects appeared either in front, to the right, to the left, or behind the participants. Once the participants' gaze vector collided with the virtual object, the object remained visible for 6 s. This procedure ensured that the same amount of time was used to encode each of the stimuli. Participants were instructed to encode these objects in detail for later recall. Based on the computational theory of mental imagery by Kosslyn and colleagues (e.g.,^36^), we divided the recall phase into an *image generation task* and an *image inspection task*. During image generation participants were cued to visualize the objects and during image inspection they evaluated a statement about a visual detail of the object (i.e., “the butterfly is red”, “there is a star on the jet”) being true or false. They were free to move within the boundaries of the virtual room during both the perceptual encoding and recall phase. However, between trials participants were forced to return to their initial position (i.e., the center of the room) and to fixate a cross which appeared in the same position across trials (i.e., straight ahead on the front wall). A schematic representation of the virtual environment during encoding and during recall is illustrated in Fig. 1. Individual differences in the processing and representation of visual information were assessed by means of the OSIQ^31^. In addition to the self-reported measurements, participants completed the image-scanning task (IST) introduced by Borst and Kosslyn^35^. The task was developed to assess individual differences in spatial imagery ability (i.e., the ability to generate, maintain, and inspect precise spatial representations).

We hypothesized higher fixation proportions during image generation and image inspection in predefined areas of interests (AOI) that correspond to the objects’ location during encoding (corresponding AOIs) compared to other possible target locations (non-corresponding AOIs) (*H1*). Furthermore, we expected that individual differences in imagery abilities moderate the difference between the corresponding AOIs and non-corresponding AOIs: Spatial visualizers (as measured with both the OSIQ spatial scale and the IST) were expected to spend more time in the corresponding AOIs compared to the non-corresponding AOIs, whereas object visualizers (as measured with the OSIQ object scale) were expected to show less looking at nothing behavior (*H2*).

## Results

To investigate individual differences in eye movements to absent objects in 3D space we presented participants with virtual objects in IVR. In a subsequent recall task, participants visualized the objects (image generation) and they were tested on their ability to recall non-spatial details (color and shape) of the objects (image inspection). We expected higher fixation proportion in the areas corresponding to the objects’ location (corresponding AOI) compared to other possible target locations (non-corresponding AOIs), depending on participant’s imagery abilities. Individual differences in imagery abilities were assessed by the OSIQ object imagery scale, the OSIQ spatial imagery scale, and the IST.

### Individual differences in imagery abilities

We found a positive correlation between the OSIQ spatial imagery scale and the IST performance, ${rho}_{Spatial:IST}=0.21;CI=\left[0.05;0.39\right]$. The 95% CI of the correlation between the OSIQ spatial imagery scale and the OSIQ object imagery scale included zero, thus indicating no correlation. The correlation matrix including the mean and standard deviation (SD) of all measurements is summarized in Table 1.

**Table 1:** Mean (SD), and matrix of correlations [l-95% CI, u-95% CI].

	Mean (SD)	(1)	(2)	(3)	(4)
**IST**					
(1) Accuracy	0.72 (0.10)	1	**0.21** [0.04, 0.38]	**0.21** [0.05, 0.39]	−0.09 [−0.25, 0.09]
(2) RT	1.74 (0.84)		1	−0.13 [−0.29, 0.06]	−0.04 [−0.21, 0.14]
**OSIQ**					
(3) Spatial	2.75 (0.66)			1	−0.13 [−0.30, 0.04]
(4) Object	3.33 (0.54)				1

(1) IST, Accuracy = Accuracy in the image-scanning task. (2) IST, RT = response time in the image-scanning task. (3) OSIQ, Spatial = mean score of the spatial imagery scale. (4) OSIQ, Object = mean score of the object imagery scale.

Estimates with credible intervals that do not include zero are in bold.

### Perceptual encoding

During encoding, participants spent on average *M* = 4.25 s (*SD* = 1.50) on the presented object, thus confirming proper encoding of the objects, which were visible for 6 s each, as soon as participants gaze collided for the first time with the object.

### Recall

#### Eye movements to absent objects and object imagery ability

**Figure 2:**
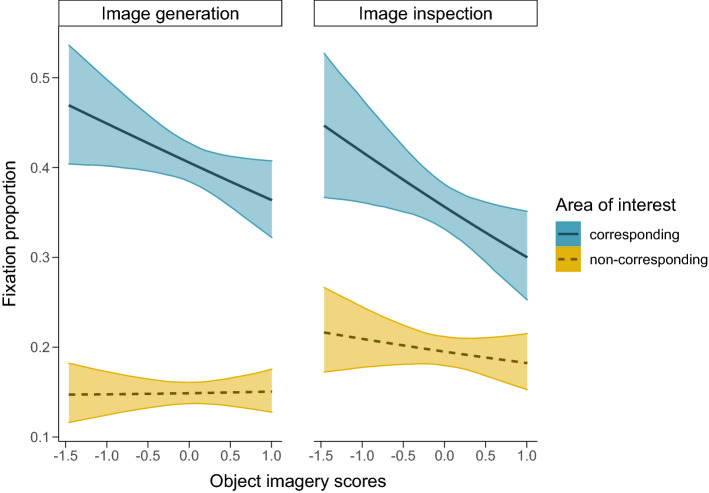
Posterior means and 95% credible intervals for the estimated fixation proportion per stimulus as a function of area of interest, task and object imagery scores (centered around the grand mean).

During recall, the mean response time per trial was M = 6.81 s (SD = 3.69) at image generation and M = 3.47 s (SD = 3.91) at image inspection. Using a Bayesian zero–one-inflated beta model, we estimated fixation proportion during the image generation and the image inspection task. The best fitting model included AOI (corresponding vs. non-corresponding), task (image generation vs. image inspection) and object imagery ability as fixed effects. The posterior draws of the beta distribution revealed a higher fixation proportion in the corresponding AOI compared to the non-corresponding AOIs, ${\beta }_{NC}=-1.37; SE=0.05;CI=\left[-1.46;-1.27\right]$. Eye movements reinstated the spatial location of previously seen objects. This effect was more pronounced during image generation compared to image inspection, ${\beta }_{NC:ImIn}=.53; SE=.07;CI=\left[.39;.68\right]$. The difference between corresponding and non-corresponding AOIs was moderated by object imagery ability, ${\beta }_{NC:Object}=.20; SE=.09;CI=\left[.03;.37\right]$. Participants with higher scores on the object imagery scale looked less at relevant locations when they generated and inspected the mental image. Model parameters are reported in Table 2 and illustrated in Fig. 2.

**Table 2:** Logit transformed regression coefficients (posterior mean, standard error, 95% credible intervals) of the continuous fixation proportion as a function of area of interest, task and object imagery scores.

	Estimate	Est.Error	l-95% CI	u-95% CI
**Group-level Effects**				
Trial (sd)	0.04	0.02	0.00	0.09
Participant (sd)	0.27	0.03	0.22	0.33
**Population-level Effects**				
Intercept	−0.38	0.05	−0.47	−0.29
phi_Intercept^1^	1.31	0.03	1.26	1.36
zoi_Intercept	0.65	0.02	0.60	0.69
coi_Intercept	−3.53	0.08	−3.69	−3.38
NC	**−1.37**	0.05	**−1.46**	**−1.27**
ImIn	**−0.20**	0.05	**−0.31**	**−0.09**
Object	**−0.18**	0.09	**−0.35**	**−0.01**
NC:ImIn	**0.53**	0.07	**0.39**	**0.68**
NC:Object	**0.20**	0.09	**0.03**	**0.37**
ImIn:Object	−0.11	0.11	−0.31	0.09
NC:ImIn:Object	−0.00	0.14	−0.29	0.27

Phi_Intercept = beta precision (dispersion) parameter (^1^log transformed). Zoi_Intercept = zero–one inflation. Coi_Intercept = conditional one inflation. NC = non-corresponding AOI. ImIn = image inspection. Object = object imagery scores. Estimates with credible intervals not including zero are indicated in bold.

#### Retrieval performance

During recall, participants correctly evaluated the statements about the visual details of the objects in 50% of all trials. Including retrieval performance in the models revealed no difference between correct and error trials regarding the looking at nothing effect, ${\beta }_{NC:Acc}=.1; SE=.09;CI=\left[-.07;.28\right]$. The 95% CI of the interaction between AOI, object imagery ability, and retrieval performance included zero, thus indicating no relation between object imagery, eye movements to absent objects with regards to retrieval performance, ${\beta }_{NC:Object:Acc}=-.03; SE=.17;CI=\left[-.36;.30\right]$. Parameters of the model including retrieval performance are provided in the Supplementary material.

#### Eye movements to absent objects and spatial imagery ability

In two additional models, spatial imagery scores (OSIQ spatial imagery scale, IST) were included instead of the OSIQ object imagery scores. Posterior draws indicated that the difference between the corresponding and the non-corresponding AOIs was not moderated by the OSIQ spatial imagery score, ${\upbeta }_{\mathrm{NC}:\mathrm{Spatial}}=.04;\mathrm{ SE}=.07;\mathrm{CI}=\left[-.09;.17\right]$ nor by performance on the IST, ${\upbeta }_{\mathrm{NC}:\mathrm{IST}}=-.2;\mathrm{ SE}=.44;\mathrm{CI}=\left[-1.08;.62\right]$. Eye movements to the location where the objects were previously encoded do not depend on individual differences in spatial imagery abilities. Parameters of the additional models (including OSIQ spatial imagery scale or IST) are provided in the Supplementary material.

#### Spatially constraint fixations

Participants did not re-fixate any of the predefined AOIs in about two thirds of the trials. The estimated probability of a binary outcome (i.e., fixating the predefined AOIs in either 0% or 100% of the entire trial duration) was 66%, $zoi\_Intercept=.65; SE=.02;CrI=\left[.60;.69\right]$. Out of these 66%, the estimated probability of fixating an AOI during the entire trial duration (coi) was 3%, $coi\_Intercept=-3.53; SE=.08;CI=\left[-3.69;-3.38\right]$. In particular, participants showed a tendency to fixate the front wall during both image generation (*M* = 69%, *SE* = 0.38) and image inspection (*M* = 69%, *SE* = 0.42). However, an additional analysis which was based on fixations to the front wall revealed that spatial information of previously inspected objects was also reflected in the spatially constraint fixations to the front wall. Furthermore, the effect was moderated by individual differences in object imagery abilities. The additional analysis is available in the Supplementary material.

## Discussion

We examined how individual differences in the processing and the representation of visual information contribute to eye movements to absent virtual objects in a 3D space. Participants tended to re-fixate the objects’ location more than other possible target locations. Thus, we replicated the looking at nothing effect in a 3D setting where participants were free to move within a restricted 3D environment. Furthermore, we demonstrated that the effect was moderated by individual differences in object imagery abilities. The higher the individuals scored on the object imagery scale, the smaller was the looking at nothing effect. It is likely that the use of a 3D environment made the impact of individual differences in object imagery more evident since previous studies were carried out on 2D screens, which narrow down the use of different strategies.

Our results support the idea that spatial location is integrated into coherent internal representations of pictorial objects. Consistent with current theories of fixation reinstatement^17,18^ and in line with previous studies on eye movements during visual imagery and visual memory, we demonstrated that spatial information is encoded and maintained automatically and that spatial location of previously seen objects biases overt attention (i.e., eye movements), even if spatial memory is not relevant for the task at hand. Further, our findings point to different integration-strategies among individuals. In agreement with previous research showing that gaze patterns during visual imagery vary significantly among individuals (e.g.,^15,26^), we found that the magnitude of the looking at nothing effect was negatively associated with object imagery ability. Individuals who are able to create and maintain clear and vivid mental representations may rely less on spatial information associated with the location of previously encoded stimuli. This supports recent findings suggesting that integrated object representations are not necessarily grounded in spatial location (e.g., when stimuli are serially presented at the same spatial location)^37^. Interestingly, and against our expectation, the magnitude of the looking at nothing effect is not associated with increased self-reported spatial imagery abilities and the IST. However, no correlations have been reported between object-based and spatial imagery scores in previous research^31,35^.

Our study design differed from previous research on looking at nothing in that we investigated eye movements to absent objects in an immersive 3D environment. Participants had to move within a virtual room to re-fixate the corresponding area, whereas in previous studies eye movement reinstatement was restricted to the surface of a 2D screen. Thus, looking back in our setting required more effort than in previous screen-based settings because of the greater distance between the initial gaze position and the target location. The reduced effect in object visualizers could be explained by a different weighting of benefits and costs of executing eye movements. For example, object visualizers process and represent visual information in terms of visual properties (e.g., shape and color)^33^ and executing eye movements might thus be less relevant for them to retrieve visual details. Given the increased probability to tap into processing costs when looking back at relevant locations in a 3D space, it is likely that object visualizers execute less eye movements to the previously visited areas but instead generate and maintain an integrated representation of the object regardless of its spatial location.

It should be noted that the looking at nothing effect in our study was not related to retrieval performance. We found similar eye-gaze patterns among correct and incorrect evaluated statements when controlling for object imagery scores, thus indicating that re-fixating the objects’ location does not facilitate retrieval of object-related features. This finding is in line with other research suggesting no beneficial effect of eye-movement reinstatement^14^, but see^8^ for an absence of the looking at nothing effect in error trials.

The spatial configuration of a scene can differ in size when it is mentally represented^26^ and previous research suggests that lower dispersed eye movements on 2D computer screens may activate the same process as when the exact same location is re-fixated^9,10^. Indeed, we found that eye movements were often spatially constraint; participants fixated the front wall (i.e., the area straight ahead) in 69% of the time. This result is in line with previous findings showing lower dispersed eye gaze patterns during visual imagery (e.g.,^1^). It is conceivable that participants in our study showed an overall tendency to execute narrow dispersed eye gaze patterns to avoid processing costs due to navigating an immersive 3D space. Thus, the looking at nothing effect might be less pronounced in real-life, because eye movement reinstatement is not restricted to a 2D screen. Yet, the looking at nothing effect was preserved in these spatially constraint fixations. Participants spent more time in the area directing towards the absent object compared to the area directing towards the opposite direction. It is interesting that the spatial correspondence of the narrow-dispersed fixations is linked to individual differences in object imagery. The eye gaze pattern in participants with lower levels of object imagery abilities reflected the spatial location of stimuli inspected earlier, whereas this was not the case for participants with higher object imagery abilities. Taken together, our results demonstrate that the looking at nothing effect in 3D space is negatively related to individual differences in object imagery ability. Individuals who tend to process and represent visual information in terms of visual properties rather than in terms of spatial layout, tend to make less eye movements to specific locations during visual imagery.

Looking at nothing was less pronounced during image inspection compared to image generation. This finding is in line with Kinjo et al. who showed that the looking at nothing effect can fade^26^. They found that the saccade rate towards the critical area peaked at 600 ms after retrieval onset. Thus, the reactivation of spatial locations may play a role early in the process of visual imagery, but not later. Once the eyes moved to the relevant location, they might follow other patterns to generate, maintain and inspect visual features of the objects. However, this remains rather speculative, as in the present study we analyzed eye movement reinstatement based on the absolute location in space. Future research is needed to investigate eye movements in a more fine-grained manner as this may provide further insight in the role of eye movements during the specific phases of the visual imagery process.

A further issue is that we found overall poorer performance in the recall task when compared to previous studies using 2D screens (e.g.,^8,38^) or to a seated IVR setting in which we used the exact same recall task^15^. Participants correctly evaluated statements about object details in 50% of the trials. A recent study by Lui et al. suggests that processing of spatial information in IVR is related to cognitive costs^39^. The authors found improved learning in a seated IVR learning experience, however not in a free moving IVR experience, when compared to a control condition (no IVR experience). In our study, participants were also free to move within the virtual room, during both encoding and recall. Thus, the precision of the memory representation may have been impaired due to increased cognitive load. However, the fact that participants fixated the corresponding areas more often than other possible target locations suggests successful retrieval of the objects. Nevertheless, future studies should vary task difficulty and consider different study designs (e.g., augmented reality) in order to disentangle memory performance from IVR specific aspects (e.g., increased cognitive load). Another point that needs further investigation is the impact of additional visual input on gaze behavior in IVR. Adding conditions with varying degrees of visual input would be important in order to investigate the relative influence of visual context on eye movements in terms of amplitude and frequency.

In summary, we provide evidence that people look back at spatial locations of previously seen objects, when they are free to move in 3D space, thus confirming ecological validity of previous findings. Furthermore, we show that the looking at nothing effect is negatively correlated with individual differences in visual object imagery. Object visualizers rely less on spatial information because they tend to process and represent the visual information in terms of color and shape rather than in terms of spatial layout. This finding suggests that eye movements may be subject to individual integration-strategies in visual imagery, depending on how individuals process and represent visual information in terms of visual properties. Moreover, we demonstrate that the combined use of IVR and eye-tracking as an effective proxy for real-life behavior provides a promising addition to conventional screen-based settings in order to investigate cognitive processes in an ecologically valid way.

## Methods

### Participants

The experiment took place at the University of Bern. A total of 85 students took part in the experiment. Participants were recruited through a participants’ recruitment portal and received course credit for participation. The results are based on 78 native German-speakers (24 males, M_age_ = 22.06, SD = 2.88, range = 18–32); data of seven participants were excluded due to technical problems. 48% of the participants reported to have no experience with IVR and the remaining 52% mentioned having used head-mounted displays once before. All participants reported normal or corrected to normal vision. Power considerations to determine the sample size was based on data simulation, indicating that a *n* = 10 was sufficient to find a small difference (0.1) in fixation proportion with a power of 0.99 (i.e., in 99% of 1000 iterations, an *n* = 10 was enough to produce intervals with a 95% probability the effect was not equal to zero). Since we were further interested in individual differences, we decided to test as many participants as possible in the period from March to December 2020 via the participant pool of the University of Bern. We expected to achieve a sample size between 70 and 100 participants. The experiment was approved by the ethics committee of the University of Bern.

### Material

#### Virtual environment and virtual objects

The virtual room including four pedestals was modeled using the 3D computer graphics software blender 2.80 (http://www.blender.org). The *x* (width), *y* (height) and *z* (depth) dimensions of the room were *x* = 500 cm, *y* = 300 cm, *z* = 500 cm and those of the pedestals were *x* = 50 cm, *y* = 100 cm, *z* = 50 cm. The pedestals were placed in front of each of the four side walls, distributed over 360 degrees. A set of 28 colored objects were gathered from online sources providing free 3D models for VR and AR. We quantified the objects maximum volume to *x* = 75 cm, *y* = 75 cm, *z* = 75 cm. Each object belonged to one of five categories (animals, sports equipment, vehicles, technical devices and characters). During the experiment the objects were presented on top of one of the four pedestals. Each object was paired with an auditory title (i.e., the name of the object) and an auditory statement about visual details (i.e., color and shape) of the object (e.g., “the bicycle is red and yellow”, “the rabbit sits on his hind legs”). Titles and statements were spoken by a female native speaker and recorded using Audacity (http://audacity.sourceforge.net). A list with stimuli title and statements is available in the Supplementary material.

#### Individual differences in imagery abilities

The OSIQ^31^ originally consists of three separate scales: an object imagery scale, a spatial imagery scale and a verbal scale. We used the object imagery and the spatial imagery scales. The 15 items of each scale were rated on a 5-point Likert scale (1 = totally disagree; 5 = totally agree). The scores were created by averaging the ratings on object imagery and spatial imagery items. Higher overall scores on the scales indicate higher values of the respective variable. The scales have shown adequate internal consistency (object imagery scale: Cronbach’s α = 0.80, spatial imagery scale: Cronbach’s α = 0.78)^35^. As regards our sample, the scales turned out to be reliable (object imagery scale: Cronbach’s α = 0.80, spatial imagery scale: Cronbach’s α = 0.86).

The IST was divided in two blocks (easy and hard). Participants were asked to decide whether an arrow was pointing to a dot based on a mental image of a four-dot pattern studied before. We created the first patterns by placing four dots, each dot being 7 mm in diameter, in a 19 cm x 19 cm white square, surrounded by a frame. The second pattern was created by rotating the first pattern by 180 degree. For each pattern, 96 arrows were created. Each arrow with a length of 2 cm long was placed at one of four possible distances (3 cm, 4.5 cm, 6 cm, 7.5 cm) from the target dot. Half of the arrows pointed directly at one of the dots and the other half missed all the dots by 20 degrees, aligned with one of the tangents of an *area of uncertainty* surrounding each dot with either a radius of 26.5 mm in the easy, or a radius of 14.5 mm in the hard block (see^35^, Experiment 3).

### Procedure

We administered the OSIQ questionnaire online 6–8 days before participants came to the laboratory using the open-source software Limesurvey (https://www.limesurvey.org/). The experiment in the laboratory was divided into two parts: the IST and the visual memory task in IVR.

*Image-scanning Task (IST).* In the IST, participant completed two blocks (easy and hard), each consisting of a learning phase and an image-scanning phase. Similar to Borst and Kosslyn’s Experiment 3 the order of the two blocks was held constant over participants^35^. That is, each participant started with the easy block.

In the *IST learning phase,* we used a print of a four-dot pattern in a 19-cm × 19-cm frame with a fixation-cross in the center. Participants were asked to memorize and then to reproduce the pattern (location of the dots) on a sheet of paper on which only the frame and the fixation-cross were shown. We printed the original pattern on a transparent sheet that participants used to compare their drawing with the actual pattern. This procedure was repeated until all dots were reproduced within 0.3 cm of their original location. Participants required 1 to 10 study-drawing cycles (*M* = 3.46, *SD* = 1.74) for appropriate memorization of the pattern.

The *IST retrieval phase* was conducted on a 24-inch monitor with resolution of 1920 × 1200 pixels and a refresh rate of 90 Hz using PsychoPy software^40^. On each of the experimental trials, participants were asked to visualize the dot-pattern they had studied before, while keeping their eyes on a fixation cross in the middle of the screen. Then, an arrow was presented in a 19 cm × 19 cm frame on screen until participants responded. Participants were prompted to decide whether the arrow was pointing on one of the dots from the previously learned four-dot pattern (Yes/No). By pressing the n-button for the decision “Yes” (i.e., the arrow points on one of the locations of the dots) and the b-button for “No” (i.e., the arrow does not point on one of the locations), using their dominant hand. The responses were self-paced, and a new trial started, when the answer was given. Prior to experimental trials participants completed 24 practice trials in which they received feedback (i.e., the dot-pattern and the arrow were presented simultaneously) after each trial. Apart from the feedback, the practice trials were identical to those in the experiment.

The *visual memory task in IVR* was rendered on an HTC Vive Pro Eye HMDs using the unreal engine 4.21.2. Eye movements were recorded at a sampling rate of 90 Hz using the HTC SRanipal SDK (https://developer.vive.com/resources/vive-sense/sdk/vive-eye-tracking-sdk-sranipal/). First, participants were informed about the upcoming procedure in IVR. After the HMD was positioned on the participant’s head, we used the five-point calibration and validation procedure provided by the SRanipal SDK.

During the *IVR encoding phase*, each trial started with the spoken title followed by the appearance of the corresponding object on one of the four pedestals. Participants were asked to thoroughly encode the objects so that they could later evaluate statements about visual details of the objects. Once the participants gaze vector collided with the object, it was visible for 6 s. The order and the spatial position of the objects (i.e., on which pedestal the object was presented) was randomized, so that the same number of objects appeared on each pedestal. During each trial, participants were free to move within the boundaries of the virtual room. In order to control for initial gaze and body position across participants and trials, a fixation cross straight ahead on the wall and a transparent blue cone (radius = 60 cm) in the center of the room was presented between trials. The cone (radius = 60 cm) extended from the floor to the ceiling of the virtual room and served as a marker for the participants' starting position in the 3D environment. Each trial was preceded by the fixation cross and the simultaneous participant’s position inside the cone for 0.5 s. Both fixation cross and cone disappeared before a new object name was presented.

The *IVR recall phase* was divided into an *image generation task* and an *image inspection task* (see^14^). During image generation participants visualized the objects and during image inspection they evaluated a statement about visual details of the objects (true/false). Each recall trial started with a cue (participants heard one of the 28 object titles), indicating the object which had to be visualized as vividly as possible. Participants pulled the trigger on the controller in their dominant hand, once they had generated the mental image. Then they heard a specific statement about the object (e.g., “the butterfly is red”) and made their response as true or false (self-paced) by pulling the trigger (in the right hand for “true”, in the left hand for “false”). As soon as participants made the true/false judgment, the trial ended. A new object name was auditorily presented, preceded by a fixation square straight ahead on the wall and participants position inside the cone for 0.5 s.

### Area of interest and data acquisition

We defined four equally sized 3D areas of interest (AOI) on top of the pedestals. The dimensions of the AOI were defined by the object’s maximal volume (*x* = 75 cm, *y* = 75 cm, *z* = 75 cm). The area where the object was presented during encoding was defined as the corresponding AOI, the other areas as non-corresponding AOIs. Data were recorded at a sampling rate of 90 Hz. Each data point was associated with the gaze origin (i.e., binocular point vector), the gaze direction (i.e., binocular direction vector) and the information whether there was an intersection of the gaze direction vector with one of the predefined areas or not.

### Analysis

Data analysis was performed in R (3.5.1; R Core Team). We followed a Bayesian approach using the *brms* package for Bayesian (non)-linear mixed models^41^ and the *bayesfactor* package for Bayesian analysis of correlations^42^. We chose Bayesian inference since it allows for estimating the relative credibility of parameters given the data (i.e., posterior probability distribution), which is not the case in a frequentist data analysis approach^43–45^. The parameters’ uncertainty was summarized by the 95% credible interval (CI). The 95% CI not containing zero indicates a 95% probability that the true (unknown) effect would be not equal to zero, given the observed data. We used Markow Chain Monte Carlo (MCMC) method implemented in *Stan* (https://mc-stan.org/) with 4 chains of 2500 iterations to calculate posterior parameter estimates. Convergence of the chains was visually inspected using trace plots. We used a stepwise approach, starting with models including all parameters in a first step. Model comparisons were based on leave-one-out cross-validation (LOO function in brms). The model that best fitted the collected data is reported. Model notations including prior distributions, the results of the model comparisons and the estimated model parameters of the entire models are available in the Supplementary material.

A fixation was defined by the visual gaze on the same AOI for at least 100 ms. That is, collisions of the gaze vector with a predefined AOI for less than 100 ms were excluded. We removed extreme values of response times (RTs > *M* + 3 × *SD* and RTs < *M* − 3 × *SD* for each participant and task—4 trials in the image generation task, 21 trials in the image inspection task).

Fixation proportion was determined as the mean dwell time in each area divided by the total time per trial. We analyzed fixation proportion by means of Bayesian generalized linear mixed models using a zero–one-inflated beta (zoib) function to handle trials with 0% or 100% fixation proportion in a given AOI. This analysis considers a beta data distribution for the continuous fixation proportion outcome in the closed (0, 1) interval. In addition, the zoib model estimates the beta precision (dispersion) parameter (phi) and considering a Bernoulli data distribution, the probability of a binary outcome (i.e., either 0% or 100% fixation proportion, zero–one-inflation, zoi) and the probability of 100% fixation proportion, given that the fixation proportion was either 0% or 100% (i.e., conditional-one-inflation, coi)^46^.

We were primarily interested in the difference between the AOIs (*H1*). In order to account for the weighted probability fixating one of the three non-corresponding areas, we calculated the mean fixation proportion of all non-corresponding areas. To estimate fixation proportion (i.e., the mean of the beta distributions) the first model thus included the fixed effect AOI comparing the corresponding AOI with the non-corresponding AOIs. We also added the fixed effect task and the interaction term between AOI and task for the difference between the image generation task and the image inspection task as well as the imagery scores (object imagery ability, spatial imagery ability, and IST), and the interaction terms between AOI, task and imagery scores for the moderations by participant’s imagery abilities (*H2*). We further included random intercepts (subject and items) to account for within-group variability. In addition, we specified the model to estimate the beta distribution’s precision (phi), the probability of a binary outcome (zoi) and the conditional one-inflation (coi).

### Ethics approval

All procedures were in accordance with the ethical standards of the institutional research committee and with the 1964 Helsinki declaration and its later amendments or comparable ethical standards.

### Informed consent

Informed consent was collected from all participants prior to the experiment.

## Supplementary Information

Supplementary Information.

## Data Availability

Data are available on OSF: https://osf.io/w75qn/ (data encoding), https://osf.io/fu25r/(data recall).
