# Supplementary material for: Imagery-related eye movements in 3D space depend on individual differences in visual object imagery
Source: Sci Rep. 2022 Aug 19;12:14136. doi: 10.1038/s41598-022-18080-4 (PMC9391428; doi:10.1038/s41598-022-18080-4)
Supplement: Supplementary file 1 — Supplementary Information. [file 41598_2022_18080_MOESM1_ESM.pdf]

Supplementary Material: Imagery-related eye movements in 3D space depend on individual differences in visual object imagery

Sandra Chiquet[1,*], Corinna S. Martarelli[2], and Fred W. Mast[1]

[1]University of Bern, Department of Psychology, Bern, 3012, Switzerland
[2] UniDistance Suisse, Faculty of Psychology, Brig, 3900, Switzerland
*sandra.chiquet@unibe.ch

The supplementary material contains additional information with regard to the analysis. We report:

1. Model formulae
2. Model Comparisons (Table S1)
3. Model parameters of the entire models (Table S2)
4. Model parameters of the model including retrieval performance (Table S3)
5. Model parameters of the additional models (Table S4 and Table S5)
6. Additional analysis
7. Stimuli and statements

Model formulae

We used the *brms* package for Bayesian (non)-linear mixed models (Bürkner, 2017). Here we provide the model formulae (1) and the prior distributions (2) for each model.

Eye movements to absent objects and imagery abilities (object imagery scores, spatial imagery scores, IST)

$$Time\ Ratio \sim 1 + AOI * Task * imagery\ abilities + (1|Trial) + (1|Participant) \quad (1)$$

$$Phi \sim 1$$

$$Zoi \sim 1$$

$$Coi \sim 1$$

$$\begin{aligned} Intercept &\sim normal(-1, 2) \quad (2) \\ \beta_{AOI} &\sim normal(0, 2) \\ \beta_{Task} &\sim normal(0, 2) \\ \beta_{ImAb} &\sim normal(0, 2) \\ \beta_{AOI\ x\ Task} &\sim normal(0, 2) \\ \beta_{AOI\ x\ ImAb} &\sim normal(0, 2) \\ \beta_{Task\ x\ ImAb} &\sim normal(0, 2) \end{aligned}$$

$$
\begin{aligned}
&\beta_{AOI\ x\ Task\ x\ ImAb} && \sim normal(0,2)\\
&\sigma_{Participant} && \sim student\_t(3,0,0.1)\\
&\sigma_{Stimulus} && \sim student\_t(3,0,0.1)\\
&Intercept_{Phi} && \sim student\_t(3,0,10)\\
&Intercept_{Zoi} && \sim logistic(0,1)\\
&Intercept_{Coi} && \sim logistic(0,1)
\end{aligned}
$$

Retrieval Performance

$$Time\ Ratio \sim 1 + AOI * Task * object * accuracy + (1|Trial) + (1|Participant) \quad (1)$$

$$Phi \sim 1$$

$$Zoi \sim 1$$

$$Coi \sim 1$$

$$
\begin{aligned}
&Intercept && \sim normal(-1,2) && (2)\\
&\beta_{AOI} && \sim normal(0,2)\\
&\beta_{Task} && \sim normal(0,2)\\
&\beta_{ImAb} && \sim normal(0,2)\\
&\beta_{Acc} && \sim normal(0,2)\\
&\beta_{AOI\ x\ Task} && \sim normal(0,2)\\
&\beta_{AOI\ x\ ImAb} && \sim normal(0,2)\\
&\beta_{Task\ x\ ImAb} && \sim normal(0,2)\\
&\beta_{AOI\ x\ Acc} && \sim normal(0,2)\\
&\beta_{Task\ x\ Acc} && \sim normal(0,2)\\
&\beta_{ImAb\ x\ Acc} && \sim normal(0,2)\\
&\beta_{AOI\ x\ Task\ x\ ImAb} && \sim normal(0,2)\\
&\beta_{AOI\ x\ Task\ x\ Acc} && \sim normal(0,2)\\
&\beta_{AOI\ x\ ImAb\ x\ Acc} && \sim normal(0,2)\\
&\beta_{ImAb\ x\ Task\ x\ Acc} && \sim normal(0,2)\\
&\beta_{ImAb\ x\ Task\ x\ ImAb\ x\ Acc} && \sim normal(0,2)\\
&\sigma_{Participant} && \sim student\_t(3,0,0.1)\\
&\sigma_{Stimulus} && \sim student\_t(3,0,0.1)\\
&Intercept_{Phi} && \sim student\_t(3,0,10)\\
&Intercept_{Zoi} && \sim logistic(0,1)\\
&Intercept_{Coi} && \sim logistic(0,1)
\end{aligned}
$$

Model comparisons

In Table 1 we report the model comparisons for the intercept-only model, the entire model (including all parameters) and the reduced model (including object imagery scores only), by showing the difference between each model and the best-fitting model respectively. The zeros in the table indicate the best-fitting model with a difference of zero with itself. Models with a smaller expected log pointwise predictive accuracy (elpd) fit the data worse.

Table S1. *Model comparison for the intercept-only model, the entire model and the reduced model.*

Analysis	Model	elpd difference	SE of the difference
Looking at nothing	intercept-only	-429.9	26.3
	entire	-8.3	3.9
	reduced	0.0	0.0

Model parameters of the entire model

Table S2. *Logit transformed regression coefficients (posterior mean, standard error, 95% credible intervals) of the entire model (fixation proportion as a function of area of interest, task, object imagery scores, spatial imagery scores, and IST).*

	Estimate	Est.Error	l-95% CI	u-95% CI
Group-level Effects				
Trial (sd)	0.04	0.03	0.00	0.10
Participant (sd)	0.28	0.03	0.22	0.34
Population-level Effects				
Intercept	-0.38	0.05	-0.47	-0.28
phi_Intercept[1]	1.30	0.03	1.25	1.35
zoi_Intercept	0.64	0.02	0.59	0.68
coi_Intercept	-3.54	0.08	-3.70	-3.38
NC	**-1.39**	0.05	**-1.48**	**-1.29**
ImIn	**-0.20**	0.06	**-0.31**	**-0.09**
Object	-0.18	0.10	-0.37	0.02
Spatial	0.08	0.07	-0.06	0.23
IST	0.30	0.48	-0.65	1.24
NC:ImIn	**0.54**	0.08	**0.39**	**0.69**
NC:Object	**0.21**	0.10	**0.01**	**0.40**
ImIn:Object	-0.08	0.12	-0.30	0.16
NC:Spatial	0.03	0.07	-0.11	0.17
ImInSpatial	0.09	0.09	-0.08	0.26
Object:Spatial	-0.03	0.13	-0.29	0.23
NC:IST	-0.06	0.47	-0.99	0.86
ImIn:IST	-0.56	0.54	-1.60	0.49

Object:IST	-0.11	0.89	-1.88	1.69
Spatial:IST	-0.82	0.66	-2.12	0.48
NC:ImIn:Object	-0.02	0.16	-0.33	0.28
NC:ImIn:Spatial	-0.15	0.12	-0.38	0.08
NC:Object:Spatial	0.09	0.13	-0.17	0.35
ImIn:Object:Spatial	-0.12	0.16	-0.43	0.19
NC:ImIn:IST	0.83	0.73	-0.59	2.26
NC:Object:IST	0.46	0.87	-1.25	2.18
ImIn:Object:IST	-0.49	0.98	-2.40	1.45
NC:Spatial:IST	1.15	0.65	-0.08	2.40
ImIn:Spatial:IST	-0.22	0.76	-1.70	1.30
Object:Spatial:IST	0.08	1.04	-2.01	2.08
NC:ImIn:Object:Spatial	0.05	0.21	-0.35	0.45
NC:ImIn:Object:IST	-0.51	1.21	-2.87	1.85
NC:ImIn:Spatial:IST	-0.42	1.01	-2.46	1.54
NC:Object:Spatial:IST	-0.17	1.00	-2.09	1.84
ImIn:Object:Spatial:IST	2.16	1.15	-0.07	4.45
NC:ImIn:Object:Spatial:IST	-0.98	1.38	-3.78	1.66

Note. Phi_Intercept = beta precision (dispersion) parameter ([1]log transformed). Zoi_Intercept = zero-one inflation. Coi_Intercept = conditional one inflation. NC = non-corresponding AOI. ImIn = image inspection. Object = object imagery scores. Spatial = spatial imagery scores. IST = Image Scanning Task. Estimates with credible intervals not including zero are indicated in bold.

Model parameters of the model including retrieval performance

Table S3. *Logit transformed regression coefficients (posterior mean, standard error, 95% credible intervals) of the continuous fixation proportion as a function of area of interest, task, object imagery scores, and retrieval performance.*

	Estimate	Est.Error	l-95% CI	u-95% CI
Group-level Effects				
Trial (sd)	0.04	0.02	0.00	0.09
Participant (sd)	0.27	0.03	0.22	0.33
Population-level Effects				
Intercept	-0.36	0.05	-0.47	-0.25
phi_Intercept[1]	1.31	0.03	1.26	1.36
zoi_Intercept	0.65	0.02	0.61	0.70
coi_Intercept	-3.54	0.08	-3.71	-3.39
NC	**-1.41**	0.06	**-1.53**	**-1.29**
ImIn	**-0.17**	0.07	**-0.32**	**-0.03**
Object	-0.15	0.10	-0.35	0.06
Acc	-0.05	0.06	-0.16	0.08
NC:ImIn	**0.48**	0.10	**0.29**	**0.69**
NC:Object	0.20	0.12	-0.03	0.44
ImIn:Object	-0.13	0.14	-0.42	0.14
NC:Acc	0.10	0.09	-0.07	0.28
ImIn:Acc	-0.09	0.11	-0.30	0.12
Object:Acc	-0.07	0.12	-0.30	0.17
NC:ImIn:Object	-0.00	0.20	-0.39	0.40
NC:ImIn:Acc	0.11	0.15	-0.18	0.39
NC:Object:Acc	-0.03	0.17	-0.36	0.30

ImIn:Object:Acc	0.12	0.21	-0.29	0.52
NC:ImInObject:Acc	-0.04	0.28	-0.59	0.51

Note. Phi_Intercept = beta precision (dispersion) parameter ([1]log transformed). Zoi_Intercept = zero-one inflation. Coi_Intercept = conditional one inflation. NC = non-corresponding AOI. ImIn = image inspection. Object = object imagery scores. Acc = accuracy in the evaluation of the statements. Estimates with credible intervals not including zero are indicated in bold.

Model parameters of the additional models (including OSIQ spatial imagery scale or performance on the IST)

Table S4. *Logit transformed regression coefficients (posterior mean, standard error, 95% credible intervals) of the continuous fixation proportion as a function of area of interest, task, and spatial imagery scores.*

	Estimate	Est.Error	l-95% CI	u-95% CI
Group-level Effects				
Trial (sd)	0.04	0.02	0.00	0.09
Participant (sd)	0.27	0.03	0.22	0.33
Population-level Effects				
Intercept	-0.39	0.05	-0.48	-0.30
phi_Intercept[1]	1.31	0.03	1.26	1.36
zoi_Intercept	0.65	0.02	0.61	0.70
coi_Intercept	-3.54	0.08	-3.70	-3.39
NC	**-1.36**	0.05	**-1.45**	**-1.27**
ImIn	**-0.21**	0.06	**-0.32**	**-0.10**
Spatial	0.08	0.07	-0.05	0.21
NC:ImIn	**0.54**	0.08	**0.39**	**0.69**
NC:Spatial	0.04	0.07	-0.09	0.17
ImIn:Spatial	0.07	0.08	-0.09	0.23
NC:ImIn:Spatial	-0.15	0.11	-0.36	0.07

Note. Phi_Intercept = beta precision (dispersion) parameter ([1]log transformed). Zoi_Intercept = zero-one inflation. Coi_Intercept = conditional one inflation. NC = non-corresponding AOI. ImIn = image inspection. Spatial = spatial imagery scores (OSIQ). Estimates with credible intervals not including zero are indicated in bold.

Table S5. *Logit transformed regression coefficients (posterior mean, standard error, 95% credible intervals) of the continuous fixation proportion as a function of area of interest, task, and IST.*

	Estimate	Est.Error	l-95% CI	u-95% CI
Group-level Effects				
Trial (sd)	0.04	0.02	0.00	0.09
Participant (sd)	0.28	0.03	0.22	0.34
Population-level Effects				
Intercept	-0.39	0.05	-0.48	-0.30
phi_Intercept[1]	1.31	0.03	1.26	1.36
zoi_Intercept	0.65	0.02	0.60	0.69
coi_Intercept	-3.53	0.08	-3.69	-3.38
NC	**-1.36**	0.05	**-1.45**	**-1.27**
ImIn	**-0.20**	0.05	**-0.30**	**-0.09**
IST	0.49	0.43	-0.35	1.33
NC:ImIn	**0.52**	0.08	**0.37**	**0.67**
NC:IST	-0.20	0.44	-1.08	0.62
ImIn:IST	-0.41	0.52	-1.42	0.63
NC:ImIn:IST	0.61	0.71	-0.80	2.01

Note. Phi_Intercept = beta precision (dispersion) parameter ([1]log transformed). Zoi_Intercept = zero-one inflation. Coi_Intercept = conditional one inflation. NC = non-corresponding AOI. ImIn = image inspection. IST = image scanning task performance. Estimates with credible intervals not including zero are indicated in bold.

Additional analysis

Given that participants showed a tendency to fixate the front wall during both image generation (M = 69%, SE = 0.38) and image inspection (M = 69%, SE = 0.42), we were further interested in whether spatial information of previously inspected objects was also reflected in this spatially constraint fixations to the front wall (i.e., local correspondence). To test whether participants looked more often in the corresponding direction, we included only those trials, where the object was previously inspected on top of the pedestal to the right or to the left. We divided the front wall by the vertical midline in two equally sized areas, which we refer to as local AOIs. The dimension of the local AOIs was x = 250 cm and y = 300 cm. The area directing towards the location where the object was inspected during encoding (i.e., the right half of the front wall when the object was presented on top of the pedestal to the right side or the left half of the front wall when the object was presented on top of the pedestal to the left side) was defined as corresponding local AOI, the other area as non-corresponding local AOI. The additional analysis was based on fixations to the front wall and on those trials in which participants did not reach the corresponding AOI, since we were interested in narrow dispersed fixations. We estimated fixation proportion using a Bayesian zero-one-inflated beta model to account for trials with 100% or 0% fixation proportion. The best fitting model included local AOI (local corresponding vs. local non-corresponding), task (generation vs. inspection), object imagery scores and the interaction terms between AOI, task and object imagery scores as fixed effects. We further included random intercepts across participants and across stimuli to account for repeated measurements. In addition, we specified the model to estimate the beta distribution's precision (phi), the probability of a binary outcome (zoi) and the conditional one-inflation (coi). Model parameters are reported in Table S6.

Table S6. *Logit transformed regression coefficients (posterior mean, standard error, 95% credible intervals) of the continuous fixation proportion as a function of local area of interest, task and object imagery scores.*

	Estimate	Est.Error	l-95% CI	u-95% CI
Group-level Effects				
Trial (sd)	0.03	0.03	0.00	0.10
Participant (sd)	0.06	0.04	0.00	0.15
Population-level Effects				
Intercept	0.17	0.05	0.08	0.27
phi_Intercept[1]	0.73	0.03	0.68	0.79
zoi_Intercept	-0.61	0.04	-0.69	-0.53
coi_Intercept	-0.59	0.07	-0.73	-0.45
LocalNC	**-0.45**	0.07	**-0.59**	**-0.31**
ImIn	-0.10	0.07	-0.25	0.05
Object	**-0.25**	0.10	**-0.44**	**-0.06**
LocalNC:ImIn	**0.41**	0.11	**0.20**	**0.62**
LocalNC:Object	**0.37**	0.14	**0.10**	**0.63**
ImIn:Object	**0.32**	0.13	**0.05**	**0.59**
LocalNC:ImIn:Object	**-0.65**	0.19	**-1.03**	**-0.27**

Note. Phi_Intercept = beta precision parameter ([1]log transformed). Zoi_Intercept = zero-one inflation. Coi_Intercept = conditional one inflation. LocalNC = local non-corresponding AOI. ImIn = image inspection. Object = object imagery scores. Estimates with credible intervals not including zero are indicated in bold.

Stimuli and statements

Stimuli	Statement
Astronaut	The astronaut is wearing a suit from NASA
Basketball	The basketball is green
Bicycle	The bicycle is yellow and red
Bouncer	The bouncer has an in-ear speaker on the left side
Boxing-gloves	The boxing gloves are from EVERLAST
Bus	The bus is handicapped accessible
Butterfly	The butterfly is red
Cat	The cat is gray
Cellphone	The time on the cell phone shows 0:00
Dog	The dog is retracting its tail
Giraffe	The giraffe pulls out his tongue
Headphones	The headphones are blue and black
Helmet	The helmet is white and blue
Horse	The hind legs of the horse are white
Jennifer (female character)	Jennifer wears a holster on her belt
Jet	There is a star on the jet
Lamborghini	The Lamborghini is black
Lamp	The lamp is white
Laptop	The laptop has a sticker to the left of the mouse pad
Ostrich	The head of the ostrich is bent down
Police car	The police car belongs to the NYPD
Rabbit	The rabbit sits on the hind legs
Robot	The robot has green eyes
Ship	The flag of the ship is yellow and blue
Soccer ball	The soccer ball is green and white
Speaker	The speaker has a red plug
Sporty man	The sporty man wears a blue tracksuit
USB stick	The USB stick stores 1 GB

Supplementary references

Bürkner, P.-C. (2017). brms: An R package for Bayesian multilevel models using Stan. *Journal of Statistical Software*, *80*(1), 1–28. https://doi.org/10.18637/jss.v080.i01
